# A comparative study of propofol alone and propofol combined with midazolam for dental treatments in special needs patients

**DOI:** 10.1097/MD.0000000000026199

**Published:** 2021-06-04

**Authors:** I-Hsin Lin, Mao-Suan Huang, Pei-Yu Wang, Ta-Sen Huang, See-Yen Chong, Sam Li-Sheng Chen, Hung-Huey Tsai

**Affiliations:** a Department of Dentistry, Taipei Medical University Shuang Ho Hospital; b School of Oral Hygiene, College of Oral Medicine, Taipei Medical University; c School of Dentistry, National Taiwan University; d Department of Dentistry, Taipei Medical University Hospital, Taipei, Taiwan.

**Keywords:** midazolam, propofol, sedation, special needs

## Abstract

Although dental treatment with sedation is performed increasingly in special needs patients, data on adding midazolam to intravenous propofol sedation are very limited for this group. The purpose of this study was to identify the factors and procedure time associated with the use of intravenous sedation with propofol alone or propofol combined with midazolam in dental patients with special needs.

This was a retrospective data analysis. The sedation medications and relevant covariates, including demographic parameters, disability levels, oral health conditions, dental procedures, treatment time, and side effects, of 718 patients with special needs were collected between April 2013 and September 2014. The unfavorable side effects by sedation types were reported. Factors associated with procedure time and the sedation medications were assessed with multiple logistic regression analyses.

Of 718 patients, 8 patients experienced unfavorable side effects (vomiting, sleepiness, or emotional disturbance) after the dental procedures; the rate was 0.6% in the 509 patients who received propofol only. In 209 patients who received propofol and midazolam, 2.4% experienced the side effects. Sedation time was associated with body mass index (BMI) < 25 (adjusted odds ratio [aOR] = 1.45, 95% confidence interval [CI]: 1.04–2.04) and the performance of multiple dental procedures (aOR = 1.44, 95% CI: 1.06–1.97) but not associated with the sedation types. A significant odds ratio for the combined use of propofol and midazolam was shown for adolescents (aOR = 2.22, 95% CI: 1.28–3.86), men (aOR = 2.05, 95% CI: 1.41–2.98), patients with cognitive impairment (aOR = 1.99, 95% CI: 1.21–3.29), and patients undergoing scaling procedures (aOR = 1.64, 95% CI: 1.13–2.39).

With the acceptable side effects of the use of propofol alone and propofol combined with midazolam, multiple dental procedures increase the sedation time and the factors associated with the combined use of propofol and midazolam are younger age, male sex, recognition problems, and the type dental procedure in the dental treatment of patients with special needs.

## 1 Introduction

The safety of health care delivery has received increasing attention in special needs dentistry recently.^[1]^ More specialized care, such as conscious sedation, may be needed for operative interventions in this particular group of patients. Due to challenges of providing treatment in this group, the caregivers should focus on providing high quality and appropriate treatment to facilitate this. Providers should make reasonable adjustments for assessing special needs patients in terms of time, equipment, and facilities.

To control pain and anxiety during dental procedures, sedation use in patients with special needs has been suggested as an alternative technology beyond local anesthesia.^[2]^ To date, conscious sedation is widely used to facilitate routine treatments in special needs patients. Intravenous sedation is considered a comfortable and effective, and it is the safest technique after inhalation sedation.^[3]^ The recovery period is shorter in comparison to that associated with general anesthesia. The most commonly used sedation medications are ketamine, propofol, and midazolam.^[4–6]^ Ketamine has an analgesic effect but may induce complications during recovery, such as severe listlessness, nausea, delirium, nystagmus, and severe muscle spasms.^[5,6]^ In contrast, propofol does not have an analgesic effect but is more likely to have the following syndromes: respiratory depression, body irritation, crying and coughing during the procedure, and anxiety without nausea during recovery.^[6,7]^ Midazolam has no analgesic effect but may induce forgetfulness after sedation.^[7]^

However, the combination of propofol and midazolam for sedation can be considered a safe, effective, and acceptable alternative to the use of propofol alone.^[8]^ However, the factors associated with medication selection and procedure time during sedation in special needs dentistry have not been adequately addressed. The aims of this study were to evaluate the factors and procedure time associated with the use of sedation medications, namely propofol and midazolam (alone or in combination), in dental patients with special needs.

## 2 Materials and methods

### 2.1 Study populations

In the present retrospective study, a total of 718 uncooperative patients with special needs, aged 12 to 100 years, who were referred to the department of special needs dentistry at Taipei Medical University Shuang Ho Hospital from April 2013 to September 2014 were enrolled. All patients underwent dental procedures at the department of special needs dentistry and received the necessary instructions for the sedative procedures before the dental procedures. Among 718 patients, 509 patients were sedated with propofol only, and 209 patients were sedated with propofol and midazolam.

### 2.2 Sedation types and patient monitoring

Sedation was achieved by 2 methods: propofol alone or propofol/midazolam. First, the dose of propofol was infused using an effect-site concentration of 50 μg/kg/min. If an adequately deep sedation level was achieved in 5 minutes, sedation was maintained using a continuous infusion of propofol throughout the procedure. If an adequately deep sedation level was not achieved after 10 minutes, the patient was given an intravenous bolus injection of midazolam (0.01 mg/kg).

Supplemental sedation according to the patient's condition (movement, phonation, etc) was allowed using a bolus injection (midazolam) or by increasing the propofol rate, if necessary. Patients were given oxygen via a nasal cannula at a rate of 2 ± 3 L/min, as needed. Heart rate, respiration, blood pressure, and oxygen saturation were monitored by the same anesthesiologist.

After the dental procedure, the patients were monitored and cared for until recovery. The patients were required to meet the following criteria before they were discharged from the hospital: the patient's vital signs (blood pressure, pulse rate, and oxygen saturation) were within the same range as their baseline values; and the patients were able to walk without staggering.

### 2.3 Data collection

The relevant factors, including demographic parameters, body mass index (BMI), disability types, oral health conditions, dental procedures, sedation types, and total procedure time, were evaluated and recorded. At the end of the procedure, each patient was monitored in the recovery room, and the conditions for recovery were also recorded.

### 2.4 Ethical consideration

The study was subsidized by government and certified as exempt from institutional review board review by the Taipei Medical University Shuang Ho Hospital, also waiving the requirement of informed consent.

### 2.5 Statistical analysis

After investigation, the data were entered into a Microsoft Excel form.

Descriptive statistics were used to explore the data. Multiple logistic regression analyses were conducted to identify the factors associated with sedation type (propofol alone or propofol/midazolam). The association between procedure time and each factor was also assessed. All statistical analyses were performed with SAS 9.4 (SAS institute, Cary, NC).

## 3 Results

Table 1 shows the descriptive statistic results (the distribution of selected variables in different age groups). The estimated results for the associations between each factor and the combined use of propofol and midazolam, while adjusting for the other factors in the model, are given in Table 2. Among demographic features, younger age was associated with a greater likelihood for the addition of midazolam. An adjusted odds ratio (aOR) (95% confidence interval [CI]: 1.28–3.86) of 2.22 was observed for teenagers, followed by adults aged 19 to 29 (aOR = 1.54 [95% CI: 0.93–2.56]). Men were more likely to receive sedation with the combination of propofol and midazolam than women (aOR = 2.05, 95% CI: 1.41–2.98). More frequent use of propofol/midazolam was noted in those who had a recognition barrier (aOR = 1.99, 95% CI: 1.21–3.29). Scaling was associated with more frequent use of propofol/midazolam (aOR = 1.64, 95% CI: 1.13–2.39).

**Table 1:** Distribution of selected variables in different age groups.

	Age					
Characteristics	12–18 N = 118	19–29 N = 205	30–39 N = 165	40–59 N = 135	≥60 N = 95	Total
Sex						
Male	77 (65%)	130 (63%)	119 (72%)	110 (81%)	49 (52%)	485
Female	41 (35%)	75 (37%)	46 (28%)	25 (19%)	46 (48%)	233
BMI						
≧25	13 (11%)	50 (24%)	47 (28%)	64 (47%)	26 (28%)	200
<25	103 (89%)	150 (76%)	118 (72%)	71 (53%)	68 (72%)	510
Sedation drugs						
Midazolam/Propofol	51 (43%)	71 (35%)	44 (27%)	23 (17%)	20 (21%)	209
Propofol Only	67 (57%)	134 (65%)	121 (73%)	112 (83%)	75 (79%)	509
Disability rating						
Mild	7 (6%)	7 (3%)	5 (3%)	3 (2%)	2 (2%)	24
Moderate	33 (28%)	28 (14%)	23 (14%)	19 (15%)	23 (25%)	126
Severe	53 (45%)	101 (50%)	64 (39%)	47 (38%)	48 (52%)	313
Extremely severe	26 (22%)	68 (33%)	71 (44%)	56 (45%)	20 (22%)	241
Cognitive impairment						
Present	98 (83%)	184 (90%)	138 (84%)	87 (64%)	81 (85%)	588
Absent	20 (17%)	20 (10%)	26 (16%)	48 (36%)	14 (15%)	128
Periodontal status						
Fair	18 (15%)	38 (19%)	33 (20%)	33 (24%)	17 (18%)	139
Poor	100 (85%)	167 (81%)	132 (80%)	102 (76%)	78 (82%)	579
No. of caries						
Edentulous	0 (0%)	0 (0%)	1 (1%)	2 (1%)	3 (3%)	6
1–5^∗^	93 (79%)	183 (89%)	143 (87%)	104 (77%)	71 (76%)	594
>6	22 (19%)	20 (10%)	17 (10%)	29 (21%)	19 (20%)	107
No caries	2 (2%)	2 (1%)	3 (2%)	0 (0%)	0 (0%)	7
No. of dental treatment procedure						
1	69 (60%)	119 (61%)	102 (63%)	89 (67%)	57 (61%)	436
≥2	45 (40%)	76 (39%)	60 (37%)	44 (33%)	93 (39%)	318
Duration of treatment						
<30 min	13 (11%)	26 (13%)	18 (11%)	23 (17%)	17 (18%)	97
31–60 min	55 (47%)	112 (55%)	77 (47%)	74 (55%)	44 (46%)	362
61–90 min	36 (30%)	51 (25%)	47 (28%)	24 (18%)	19 (20%)	177
91–120 min	9 (8%)	12 (6%)	17 (10%)	7 (5%)	9 (9%)	54
>121 min	5 (4%)	4 (2%)	6 (4%)	7 (5%)	6 (6%)	28

**Table 2:** Factors affecting the usage of additional medicine (midazolam).

	Univariate		Multivariate	
Variables	OR	*P*-value	Adjusted OR	*P*-value
Age				
12–18	2.18 (1.28–3.7)		2.22 (1.28–3.86)	
19–29	1.51 (0.94–2.45)	<.0001	1.54 (0.93–2.56)	<.0001
30–39	1.04 (0.62–1.74)		1.08 (0.63–1.87)	
40–59	0.59 (0.33–1.06)		0.7 (0.37–1.31)	
60+	1.00		1.00	
Sex				
Male	1.81 (1.27–2.58)	.001	2.05 (1.41–2.98)	.0002
Female	1.00		1.00	
BMI				
≧25	1.2 (0.85–1.7)	.3023		
<25	1.00			
Disability rating				
Severe	1.35 (0.92–1.98)	.1312	1.23 (0.82–1.86)	.3168
Extremely Severe	0.59 (0.38–0.92)	.0193	0.55 (0.34–0.88)	.0118
Absent/Mild/Moderate	1.00		1.00	
Number of caries				
>6 teeth/edentulous	0.72 (0.46–1.14)	.1601		
1–5 teeth/no caries	1.00			
Periodontal condition				
Poor	1.46 (0.97–2.18)	.0669		
Fair	1.00			
Cognitive impairment				
Present	1.88 (1.19–2.98)	.0072	1.99 (1.21–3.29)	.007
Absent	1.00		1.00	
Number of procedures				
≥2	1.31 (0.95–1.81)	.1062		
1	1.00			
Treatment				
Scaling	1.71 (1.2–2.44)	.0029	1.64 (1.13–2.39)	.01
Fillings	1.37 (0.94–1.98)	.0983	1.22 (0.83–1.81)	.3149
Root canal treatment	1.17 (0.68–2.01)	.5813	1.15 (0.65–2.03)	.6398
Extraction	1.51 (0.98–2.34)	.062	1.52 (0.96–2.42)	.0757
Prosthodontics	1.36 (0.56–3.3)	.4937	1.3 (0.49–3.42)	.6009
Periodontal treatment	1.4 (0.77–2.53)	.2739	1.6 (0.85–3)	.1453
Others	1.00		1.00	

Table 3 shows the estimated results regarding the associations between each factor and the time for carrying out the dental procedure. BMI < 25 (aOR = 1.45, 95% CI: 1.04–2.04) and multiple dental procedures (aOR = 1.44, 95% CI: 1.06–1.97) were associated with increased sedation time. A longer time was needed for endodontic therapy (aOR = 1.53, 95% CI: 0.85–2.74). The sedation types were not associated with the procedure time (OR = 1.06, 95% CI: 0.74–1.53), although to the procedure time tended to increase with the use of the combined drugs.

**Table 3:** Analysis of factors affecting the procedure time (>1 hour).

	Univariate		Multivariate	
Variables	OR	*P*-value	Adjusted OR	*P*-value
Age				
12–18	0.95 (0.58–1.56)			
19–29	0.63 (0.4–0.98)	.0437		
30–39	0.95 (0.6–1.5)			
40–59	0.51 (0.3–0.84)			
60+	1.00			
Sex				
Male	1.04 (0.76–1.43)	.7897		
Female	1.00			
BMI				
<25	1.45 (1.04–1.24)	.0317	1.45 (1.04–1.24)	.0358
≥25	1.00		1.00	
Disability rating				
Severe	0.65 (0.45–0.94)	.0227		
Extremely severe	0.72 (0.49–1.06)	.1003		
Absent/Mild/Moderate	1.00			
Number of caries				
>6 teeth/edentulous	1.45 (0.98–2.15)	.0641		
1–5 teeth/no caries	1.00			
Periodontal condition				
Poor	1.07 (0.75–1.54)	.7029		
Fair	1.00			
Cognitive impairment				
Present	1.02 (0.69–1.50)	.9350		
Absent	1.00			
Number of procedures				
≥2	1.44 (1.06–1.97)	.0195	1.44 (1.06–1.97)	<.0001
1	1.00		1.00	
Treatment				
Scaling	0.56 (0.4–0.79)	.0011	0.15 (0.08–0.26)	<.0001
Fillings	1.26 (0.87–1.81)	.2166	0.45 (0.27–0.73)	.0012
Root canal treatment	3.59 (2.16–5.97)	<.0001	1.53 (0.85–2.74)	.1569
Extraction	0.78 (0.5–1.2)	.2574	0.3 (0.17–0.51)	<.0001
Prosthodontics	0.28 (0.09–0.84)	.0239	0.15 (0.05–0.46)	.0009
Periodontal treatment	2.47 (1.42–4.31)	.0014	0.66 (0.33–1.34)	.2504
Others	1.00		1.00	
Drugs in sedation				
Midazolam/Propofol	0.92 (0.67–1.28)	.6304	1.06 (0.74–1.53)	.7521
Propofol Only	1.00		1.00	

Of 718 patients, 8 cases of unfavorable side effects (vomiting, sleepiness, or disturbed) after the dental procedures were observed. A total of 2.4% (5/209) of patients who received the combination of propofol and midazolam experienced side effects, which was higher than the 0.6% (3/509) in patients who received propofol only. Men had a higher rate of side effects (1.2%, 6/485) than women (0.9%, 2/238). Of the 8 patients with side effects, 4 patients were aged 19 to 29 years (Table 4). The rate of side effects was high among patients with treatment times between 91 and 120 minutes (3.7%, 2/54), followed by patients with a treatment time between 31 and 60 minutes (5/362, 1.4%).

**Table 4:** Side effects after the dental procedures.

No.	Sedation type	Age	Sex	Treatment time	Treatments
1	Propofol only	14	M	Less than 30 min	Scaling
2	Propofol only	25	M	91–120 min	Extraction
3	Propofol only	45	F	31–60 min	Extraction
4	Propofol/midazolam	21	M	31–60 min	Scaling/Cavity filling
5	Propofol/midazolam	25	M	31–60 min	Endodontic
6	Propofol/midazolam	21	M	31–60 min	Scaling/Cavity filling
7	Propofol/midazolam	63	F	31–60 min	Scaling
8	Propofol/midazolam	30	M	91–120 min	Cavity filling/extraction

## 4 Discussion

The main objective of the present study was to investigate the factors associated with the use of intravenous sedation with propofol alone or in combination with midazolam by examining the disability level, oral health condition, treatments, and background of patients with cognitive impairment seeking dental care at a special needs dentistry facility. Regardless of the type of sedation used, according to the results of this study, both sedation types (propofol only or in combination with midazolam) seem to be supported as effective and safe technologies when treating patients with special needs.^[9,10]^ The side effects were observed in only 1.1% of the dental procedures after sedation. The findings were consistent with previous study conducted by Reinhart et al^[11]^ that showed 0.96% of adverse event rate in outpatients with general anesthesia using propofol or in combination with midazolam. A slightly increase in unfavorable side effects was observed with the use of propofol/midazolam compared with the use of propofol only. It should be noted that men, patients who underwent multiple dental procedures, and patients who received multiple sedation drugs were more likely to have side effects. Although previous studies have indicated that unfavorable side effects are small,^[11–14]^ the absolute percent of side effects by sedation type was first reported in this study based on our large clinical surveillance.

We found that 29% of our special needs dentistry patients received sedation with propofol plus midazolam. Forty-three percent of the combined use of propofol and midazolam was found in teenage patients, followed by 35% fin patients aged 19 to 29, 27% in patients aged 30 to 39, and 17% in patients aged 40 to 59. Younger patients were more likely to receive propofol combined with midazolam. One of the reasons for this phenomenon could be the greater likelihood of restlessness in younger patients.^[15]^ Other explanations for the combined use of propofol and midazolam for sedation are related to factors such as oral health condition and dental treatments. The disease burden for patients with special needs was generally high. The proportion of poor periodontal status was approximately 80% in our investigation. In a previous study of oral health investigations in adults with intellectual and developmental disabilities, the prevalence of periodontitis was 80.3%.^[16]^ The disease burden of periodontal disease is comparable to that reported in the previous study. Moreover, the young patients had worse periodontal status and received more dental procedures in our study. As the study setting was a first demonstration center of special needs dentistry in Taiwan, the patients seen at this facility, particularly the younger patients, are not only prevalent patients but also patients seeing treatment that will accommodate their need for sedation. Therefore, the younger patients had worse oral health conditions when they were firstly treated with sedation. Therefore, the longer treatment times that accompany the selection of drug combinations for use in the treatments is acceptable.

In addition, sex, intelligent disability level, and the type of dental procedure showed statistically significant differences between the 2 groups: propofol alone or propofol combined with midazolam. In the present retrospective study, men with severe intelligent disability and patients who underwent scaling were more likely to use the propofol/midazolam combination. The underlying reasons might also be associated with restlessness, the sedative agents, and the expected procedure time. The decision regarding sedation type might be impacted by several factors. For example, the combined use of propofol and midazolam is more likely to be selected when the scaling procedure is scheduled because the procedure might have a longer expected treatment time than other dental procedures. However, further investigation is needed.

The procedure-related time was highly dependent on the type of dental procedure and number of dental procedures. A longer procedure time is required for multiple dental procedures and certain special dental procedures. However, the use of multiple sedation drugs was not associated with the procedure time. A previous study also demonstrated that the use of intravenous conscious sedation with propofol only versus midazolam with propofol did not differ with regard to treatment time in general dental patients.^[14]^

This study has some limitations. First, the procedures were not designed according to the objective of the study because this was a retrospective study. Indication bias might exist in some findings. Confounding by indication might appear when sedation type selection is associated with the outcome of interest. As our data were obtained from a single hospital, the results might not be able to generalize to patients at other centers. Second, the reaction during sedation and the recovery time after sedation was not recorded. Third, some variables, including vital sign changes during sedation, recovery time, other detailed adverse events, and visual analog scales measuring patient tolerance in relation to the efficacy of sedation, were not included in the analysis. These factors might be helpful for guiding the delivery of intravenous sedation.

In conclusion, the factors associated with the combined use of propofol and midazolam are younger age, male sex, recognition problems, and the type dental procedure. Multiple dental procedures increase the sedation time in patients with special needs. An individualized anesthetic plan and management and teamwork between the dentist and anesthesiologist are key points to materialize safe, successful, and satisfactory anesthetic conduct for special needs patients. The findings in this study are helpful for recommendations regarding intravenous sedation drug use in special needs dentistry.

## Acknowledgment

The authors are grateful to Ministry of Health and Welfare, Taiwan for providing administrative and financial support.

## Author contributions

**Conceptualization:** I-Hsin Lin, Mao-Suan Huang, See-Yen Chong, Hung-Huey Tsai.

**Data curation:** Ta-Sen Huang.

**Formal analysis:** Pei-Yu Wang, See-Yen Chong.

**Methodology:** Pei-Yu Wang, Ta-Sen Huang, Hung-Huey Tsai, Sam Li-Sheng Chen.

**Project administration:** Mao-Suan Huang.

**Supervision:** Hung-Huey Tsai.

**Writing – original draft:** I-Hsin Lin, Mao-Suan Huang.

**Writing – review & editing:** Hung-Huey Tsai, Sam Li-Sheng Chen.
